# Identification of TNFAIP8 as an Immune-Related Biomarker Associated With Tumorigenesis and Prognosis in Cutaneous Melanoma Patients

**DOI:** 10.3389/fgene.2021.783672

**Published:** 2021-12-01

**Authors:** Yuliang Sun, Jianxiong Zhao, Xiaoru Sun, Guangxin Ma

**Affiliations:** ^1^ Department of Orthopedics, Qilu Hospital of Shandong University, Jinan, China; ^2^ Department of Hand Surgery, Qilu Hospital of Shandong University, Jinan, China; ^3^ Key Laboratory of Experimental Teratology, Department of Human Anatomy, Ministry of Education, Shandong University School of Medicine, Jinan, China; ^4^ Department of Biostatistics, School of Public Health, Cheeloo College of Medicine, Shandong University, Jinan, China; ^5^ Hematology and Oncology Unit, Department of Geriatrics, Qilu Hospital of Shandong University, Jinan, China

**Keywords:** TNFAIP8, immune infiltration, SKCM, biomarker, prognostic value

## Abstract

Tumor necrosis factor-α–induced protein 8 (TNFAIP8) is a member of the TIPE/TNFAIP8 family which is associated with inflammation and tumorigenesis. The potential role of TNFAIP8 in a tumor immune microenvironment in skin cutaneous melanoma (SKCM) has not yet been investigated. The TNFAIP8 expression was evaluated *via* gene expression profiling interactive analysis (GEPIA). We also evaluated the influence of TNFAIP8 on overall survival *via* GEPIA and PrognoScan. After GO and KEGG pathway analyses, the correlation between the TNFAIP8 expression level and immune cells or gene markers of the immune infiltration level was explored by R-language. The result showed the TNFAIP8 expression was significantly reduced in SKCM in comparison with normal control. In SKCM, the TNFAIP8 expression in higher levels was associated with the better overall survival. The high expression of TNFAIP8 was positively correlated with the immune score and promoted immune cell infiltration in SKCM patients. TNFAIP8 can be a positive prognosis marker or new immunotherapy target in SKCM.

## Introduction

Skin cutaneous melanoma (SKCM) is one of the most fatal types of skin cancer (Siegel et al., 2020). Due to the high rate of invasion and distant metastasis, SKCM accounts for 72% of skin cancer mortality (Schadendorf et al., 2018). In recent years, immune checkpoint blockade has showed remarkable clinical benefits in lymphoma, melanoma, and NSCLC (Robert et al., 2015; Weber et al., 2015; Weber et al., 2017). Immune checkpoint blockade is generally effective for a wide range of cancer types, and most are not restricted by certain gene mutation status (Curti and Faries, 2021). The use of immunotherapy in SKCM has shown approximately 50% improvement in recurrence-free survival (Curti and Faries, 2021), fully revealing the regulatory roles in the mechanism of immune homeostasis are of great clinical application potential.

Tumor necrosis factor-α–induced protein 8 (TNFAIP8) is a member of the TIPE/TNFAIP8 family, which was first found in human head and neck squamous cell carcinoma (Patel et al., 1997), and play important roles in immunity, oncogenesis, and tumor progression (Lou and Liu, 2011). Its aberrant expression has been successfully validated in multiple cancer types (Zhang et al., 2018). TNFAIP8 is highly expressed in immune organs such as the lymphatic system, spleen, thymus gland, thyroid, bone marrow, and placenta, while solid organs show relatively lower TNFAIP8 expression (Lou and Liu, 2011). Few data further shed light on TNFAIP8 in immune response processes, including an increasing trend of TNFAIP8 mRNA levels in tumor-infiltrating CD4^+^ CD8^+^ T cells (Duan et al., 2014) and the tendency of CD4^+^ T cells immune function modulation both in mice and humans (Chu et al., 2003; Xin et al., 2016). Although TNFAIP8 has shown its ability in oncogenesis and immune regulation, evidence on the clinical value and distinct function of the TNFAIP8 protein in SKCM remains unveiled.

In this study, we present the expression of TNFAIP8 and its prognostic value on the overall survival (OS) in SKCM. We explored the differential expression of TNFAIP8 in tumor and normal tissues in multiple cancer types to evaluate the impacts of TNFAIP8 on different tissues. The Cox regression model was used to test the association of TNFAIP8 on OS in SKCM. The related proteins and genes of TNFAIP8 were analyzed by gene set enrichment analysis (GSEA). Furthermore, we explored the correlation between the TNFAIP8 expression and immune infiltration immune cell–related markers in the tumor microenvironment by bioinformatics tools. We unveiled the impact of TNFAIP8 on CD4^+^ CD8^+^ T cells in SKCM. Taken together, we provide evidence that decreased TNFAIP8 expression could be a novel poor prognostic biomarker for SKCM patients, and its correlation with immune cells might provide new insights into investigating the mechanism of SKCM.

## Materials and Methods

### Data Collection

**FIGURE 1:**
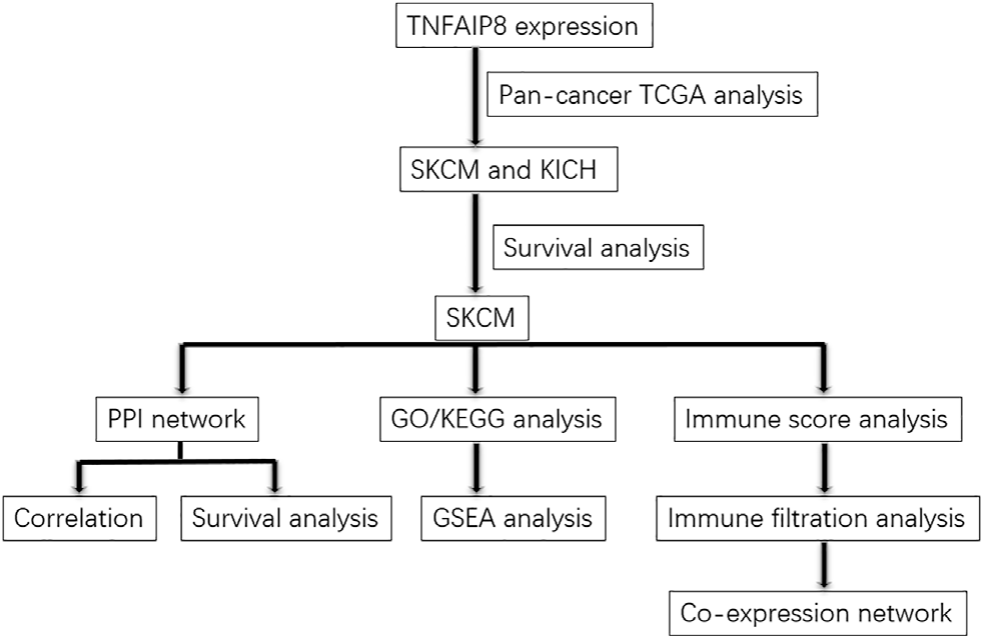
Bioinformatics workflow of this study.

The expression data of TNFAIP8 of 456 SKCM tumor samples and the corresponding clinical data were collected from The Cancer Genome Atlas (TCGA) database (https://portal.gdc.cancer.gov/). The samples with the missing survival status or with no survival time were excluded. This study has been approved by the Ethics Committee of Qilu Hospital (Jinan, China). The data were retrieved from published literature, and all analyses were performed in accordance with the Declaration of Helsinki. Figure 1 demonstrates the flowchart for the analyses of the association between the expression of TNFAIP8 and the overall survival of SKCM patients.

### Analyses of TNFAIP8 in Multiple Cancer Types

The GEPIA (http://gepia.cancer-pku.cn) platform can be used to analyze differential expression profiles associated with various types of tumors in TCGA and the Genotype-Tissue Expression Project (GTEx) (http://www.gtexportal.org/home/index.html) databases, incorporating RNA-seq expression data for 9,736 tumor samples and 8,587 control samples. Here, GEPIA was used for distinct TNFAIP8 expression and its impact on OS in multiple cancer types. It was also used to observe the correlation between TNFAIP8 expression and immune check point markers. To further explore the expression difference of TNFAIP8, the expression levels of TNFAIP8 between tumors [461 samples for SKCM and 66 samples for kidney chromophobe (KICH) in TCGA] and normal tissues (558 samples for SKCM and 53 samples for KICH in GTEx) in melanoma were explored.

### The Survival Analysis of TNFAIP8 in SKCM and KICH

PrognoScan (http://dna00.bio.kyutech.ac.jp/PrognoScan/) was used for survival analysis (Mizuno et al., 2009). GSE19234 dataset was selected to study the correlation between TNFAIP8 expression and the DFS in SKCM.

### The Integrated Protein–Protein Interaction Analysis of TNFAIP8

To illustrate the function of TNFAIP8 in tumorigenesis, we constructed an integrated protein–protein interaction (PPI) network *via* GeneMANIA (http://www.genemania.org) to screen out the targeting TNFAIP8-binding proteins and possible TNFAIP8-related genes (Franz et al., 2018). The network depicting genes functionally close to TNFAIP8 were illustrated by recognizing patterns of gene co-annotation in the gene ontology or by using enrichment analysis. Terms with both *p* value < 0.01 and FDR <0.05 were considered significantly enriched.

### Gene Set Enrichment Analysis of TNFAIP8

Gene set enrichment analysis (GSEA) was also conducted using the online tool DAVID v6.8 (https://david.ncifcrf.gov/) and LinkedOmics (http://www.linkedomics.org/admin.php). The DAVID v6.8 (https://david.ncifcrf.gov/) database was utilized to conduct Kyoto Encyclopedia of Genes and Genomes (KEGG) and gene ontology (GO) analyses of TNFAIP8 (Huang et al., 2007). Moreover, GO enrichment analysis was used to assess putative biological processes (BP), molecular functions (MFs), and cellular components (CC) associated with genes of interest. KEGG analysis defined the pathways associated with TNFAIP8 function and co-expression genes. Analyses utilized the human genome as a background parameter.

LinkedOmics is an open access portal that contains the multi-omics data of 32 TCGA cancers (Vasaikar et al., 2018). TCGA-SKCM was selected as the interested cancer cohort, for which the RNAseq datatype was selected as the search dataset and target dataset. The expression dataset of 6,843 genes related to the expression of TNFAIP8 was used to perform GSEA using the “LinkInterpreter” module. The top-ranking enrichment term for TNFAIP8 was shown by rank criteria of statistics. R package apcluster was used to cluster gene sets.

### Functional Enrichment Analysis

The DAVID v6.8 (https://david.ncifcrf.gov/) database was utilized to conduct Kyoto Encyclopedia of Genes and Genomes (KEGG) and gene ontology (GO) analyses of TNFAIP8 (Huang et al., 2007). Moreover, GO enrichment analysis was used to assess putative biological processes (BP), molecular functions (MFs), and cellular components (CC) associated with genes of interest. KEGG analysis defined the pathways associated with TNFAIP8 function and co-expression genes. Analyses utilized the human genome as a background parameter. Terms with both *p* value < 0.01 and FDR <0.05 were considered significantly enriched.

### Association Between the Tumor Microenvironment and TNFAIP8

Based on the Estimation of STromal and Immune cells in MAlignant Tumor tissues using Expression data (ESTIMATE) algorithm (Yoshihara et al., 2013), the immune and stromal scores in TME were obtained using the R package estimate. All patients were classified into two groups based on the mediation of immune and stromal scores. Cox regression models were used to test the differences between survival probabilities in high and low groups of immune/stromal scores. *p* value <0.05 was considered significant.

The CIBERSORT package was used to calculate the proportion of lymphocyte infiltration in 474 patients with SKCM in TCGA. The Ggplot2 package was used to plot the correlation between the TNFAIP8 expression level and the proportion of each lymphocyte. *p* value <0.05 was considered significant. In the whole study, *p* values were corrected using the Benjamini–Hochberg method to control the FDR for multiple testing when appropriate.

## Results

### Clinical Characteristics of the Study Patients

Table 1 summarizes the detailed clinical characteristics of 456 SKCM samples in both TNFAIP8-high and TNFAIP8-low groups from TCGA. We found SKCM patients in the TNFAIP8-high group have a higher percentage of the metastasis tumor type. Moreover, in comparison to the other group, high TNFAIP8 expressed group included higher stage I and lower stage IV percentage. Additionally, the TNFAIP8-high group was with a higher overall survival by the end of the cohort.

**TABLE 1:** Description of clinical characteristics for 456 SKCM samples in both TNFAIP8-high and TNFAIP8-low groups from TCGA.

Clinical characteristic	TNFAIP8-low group (*n* = 228)	TNFAIP8-high group (*n* = 228)	Total (*n* = 456)	*p* Value
Age (y)	59.68 ± 15.92	56.72 ± 15.46	58.2 ± 15.74	0.045
Gender				
Male	149 (65.64)	134 (58.77)	283 (62.20)	0.157
Female	78 (34.36)	94 (41.23)	172 (37.80)	
BMI (kg/m^2^)	27.28 ± 5.54	29.13 ± 6.72	28.08 ± 6.13	0.02
Sample type				
Metastatic	161 (70.93)	192 (84.21)	353 (77.58)	0.001
Primary tumor	66 (29.07)	36 (15.79)	102 (22.42)	
Tumor stage				
I	30 (14.63)	48 (23.53)	78 (19.07)	0.019
II	81 (39.51)	57 (27.94)	138 (33.74)	
III	80 (39.02)	90 (44.12)	170 (41.56)	
IV	14 (6.83)	9 (4.41)	23 (5.62)	
Neoadjuvant				
Yes	17 (7.49)	7 (3.07)	24 (5.27)	0.058
No	210 (92.51)	221 (96.93)	431 (94.73)	
Vital				
Alive	106 (46.70)	131 (57.71)	237 (52.20)	0.024
Dead	121 (53.30)	96 (42.29)	217 (47.80)	

*Data are represented as mean ± SD and frequency (percent) for numeric and category variables, respectively.

### Differential Expression of TNFAIP8 in Multiple Cancer Types

**FIGURE 2:**
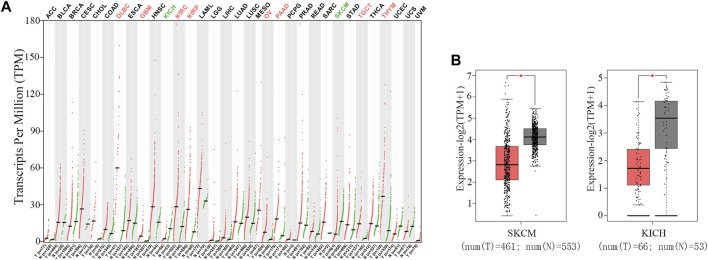
Expression of TNFAIP8 in both tumor and normal tissues of multiple cancer types. **(A)** The mRNA expression of the TNFAIP8 expression profile across all tumor samples (red dots) and paired normal tissues (green dots). The *y*-axis at the top shows the names of the cancer types (red indicates that TNFAIP8 is upregulated in tumor samples, green indicates downregulated, and black indicates non-significance). The *y*-axis at the bottom shows the sample size of tumor and normal tissues in each cancer. **(B)** The mRNA expression of TNFAIP8 in SKCM and KICH samples (tumor samples in red from TCGA, and normal samples in gray from GTEx). *p < 0.01.

To explore the differential expression of TNFAIP8 in tumor and normal tissues of multiple cancer types, the web server GEPIA was used (Tang et al., 2017). TNFAIP8 expressions were higher than adjacent tissues in many cancer types, including lymphoid neoplasm diffuse large B-cell lymphoma, glioblastoma multiforme, kidney renal clear cell carcinoma, kidney renal papillary cell carcinoma, ovarian serous cystadenocarcinoma, pancreatic adenocarcinoma, testicular germ cell tumors, and thymoma (Figure 2A). This is in line with the evidence that TNFAIP8 acts as antiapoptotic and pro-oncogenic signaling molecules and plays important roles in oncogenesis and tumor progression (Lou and Liu, 2011). However, its expression level in KICH and SKCM showed a significant reduction compared to normal tissues (Figure 2B).

### The Prognostic Value of TNFAIP8 in SKCM and KICH

**FIGURE 3:**
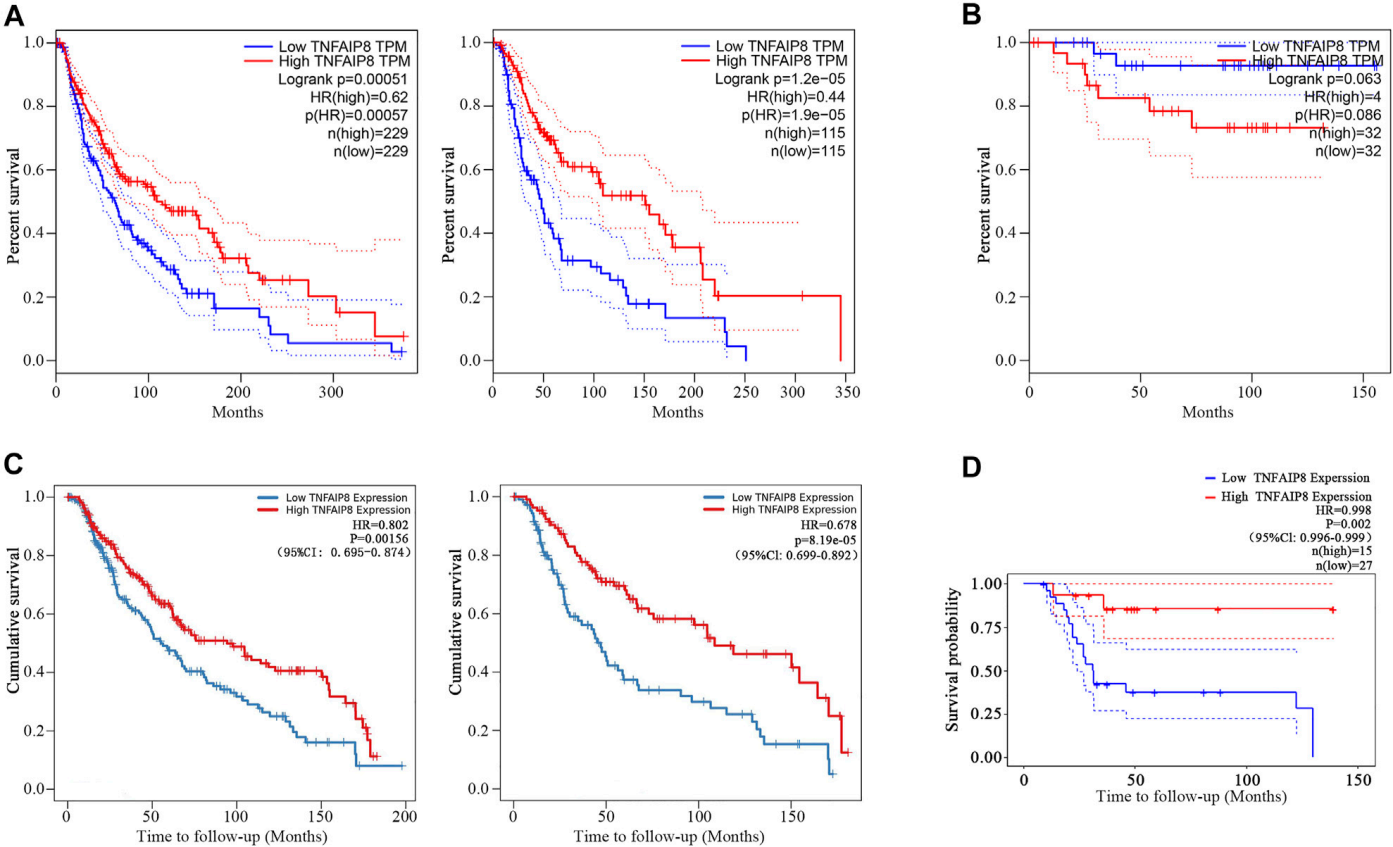
Prognostic value of TNFAIP8. **(A)** The overall survival analyses of TNFAIP8 in SKCM *via* GEPIA. Left: median cutoff group; right: quartile cutoff group. **(B)** The overall survival analyses of TNFAIP8 in KICH *via* GEPIA. **(C)** The impact of TNFAIP8 mRNA expression difference on overall survival in both SKCM (left) and SKCM metastasis patients (right) *via* the TIMER online platform. **(D)** Comparing the high and low expression levels of TNFAIP8 in skin melanoma patients with Kaplan–Meier OS curves by PrognoScan (GSE19234).

We further explored the impacts of TNFAIP8 on the overall survival (OS) of SKCM and KICH patients in TCGA. We found that the low TNFAIP8 expression was correlated with poor OS in both the median and quartile cutoff groups in SKCM (Figure 3A), which indicated an anti-oncogenic potential of TNFAIP8 in SKCM. However, the TNFAIP8 expression revealed no significance on OS in KICH (Figure 3B). Thus, we decided to focus on the association between the TNFAIP8 expression and OS in SKCM for subsequent analyses.

Furthermore, in the Cox analysis of TNFAIP8 using the tumor immune estimation resource (TIMER) (Li et al., 2016), we found that higher TNFAIP8 expressions were also dramatically consistent with the optimistic OS in both SKCM and SKCM metastasis patients (*p* = 0.002 and *p* < 0.001) (Figure 3C). Comparing the high and low expression levels of TNFAIP8 in skin melanoma patients with Kaplan–Meier OS curves by PrognoScan (GSE19234), high TNFAIP8 expression levels were also corresponded with good OS prognosis in melanoma (*n* = 42, *p* = 0.002) (Figure 3D). These results led that TNFAIP8 can be recognized as a prognostic marker for SKCM patients.

### TNFAIP8 Interacts With PAICS and PTPRC

**FIGURE 4:**
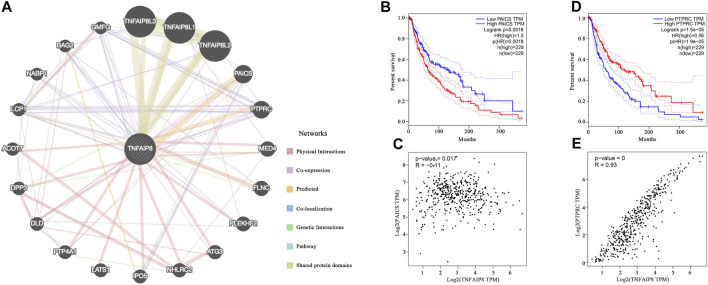
Protein–protein interaction network prediction of TNFAIP8. **(A)** Integrated protein–protein interaction network of TNFAIP8 *via* GeneMANIA. **(B)** The overall survival analyses and **(C)** TNFAIP8 correlation of PTPRC in SKCM (GEPIA). **(D)** The overall survival analyses of PAICS in SKCM *via* GEPIA. **(E)** The correlation analysis between TNFAIP8 and PAICS.

To illustrate the function of TNFAIP8 in tumorigenesis, we constructed an integrated PPI network *via* GeneMANIA to screen out the targeting TNFAIP8-binding proteins. Besides TNFAIP8L1/2/3 which belongs to the same family and share protein domains, PAICS and PTPRC were predicted to be the most possible proteins that interacted with TNFAIP8 (Figure 4A). Phosphoribosylaminoimidazole carboxylase, phosphoribosylaminoimidazole succinocarboxamide synthetase (PAICS) is an enzyme of the purine biosynthesis pathway and correlated with upregulated cancer phenotypes such as proliferation and invasion in multiple cancer types (Agarwal et al., 2020). In SKCM, although the correlation was not that high, PAICS showed a low but significant negative correlation with TNFAIP8 (*p* = 0.017, *R* = −0.11) and a high PAICS expression, indicating poor OS, with *p* = 0.0016 (Figures 4B,C). Protein tyrosine phosphatase, receptor type C (PTPRC, CD45), dominantly regulates T and B lymphocyte activation, development, tolerance, and survival. Altered PTPRC expression leads to immune suppression including T- and B-cell dysfunction and weakened immune cell adhesion and migration and severe combined immunodeficiency (Rheinländer et al., 2018). High PTPRC stood for poor prognosis in the SKCM TCGA cohort (Figure 4D). The expressions of TNFAIP8 were positively correlated (Figure 4E, *p* < 0.001, *R* = 0.93). These conclusions suggest the potential role of TNFAIP8 in SKCM tumor development and immune regulation.

### GO and KEGG Pathway Analyses of TNFAIP8 in SKCM

**FIGURE 5:**
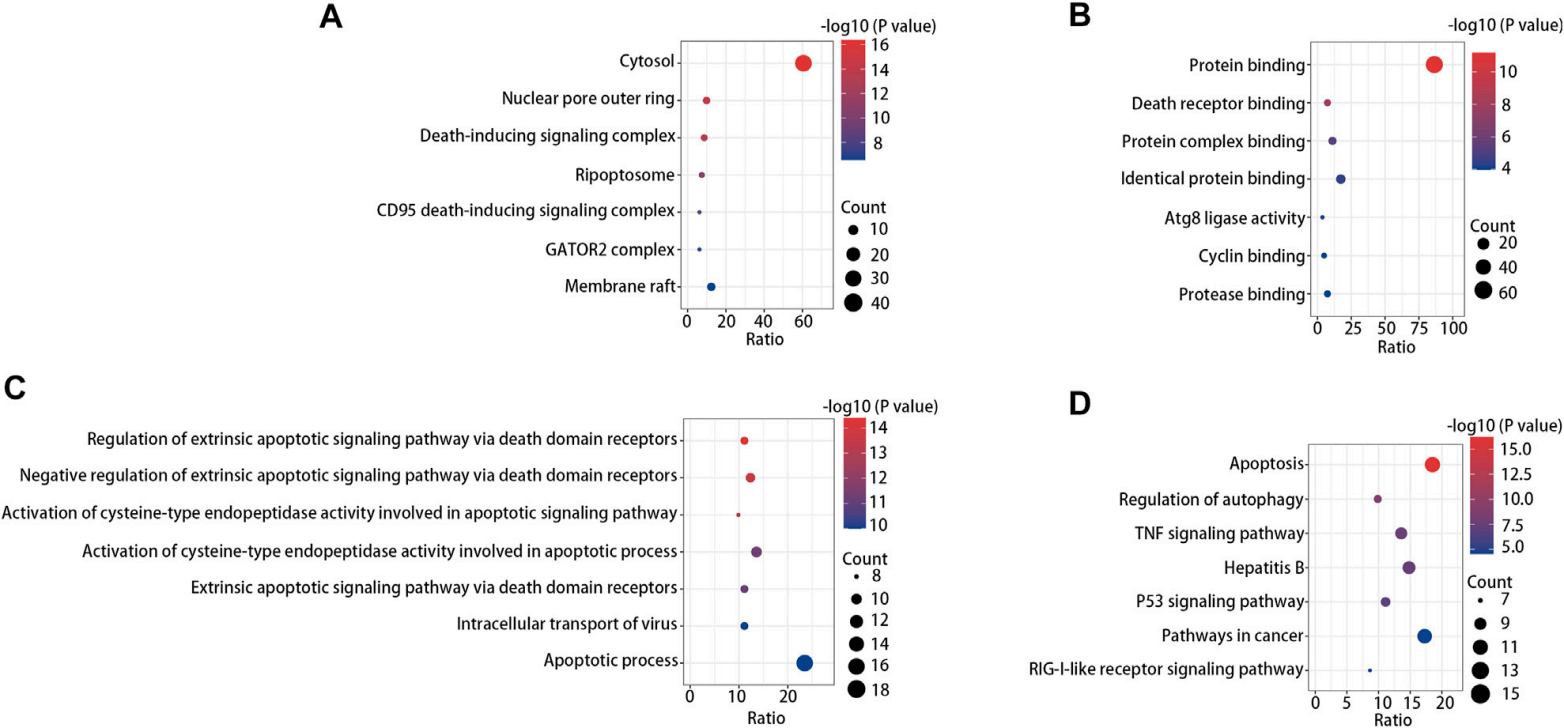
GO enrichment analysis result of cellular component, **(A)** molecular function, **(B)** and biological process **(C)**. **(D)** Top seven enriched KEGG pathway distribution.

After PPI prediction, GO enrichment and KEGG pathway analyses were performed for further TNFAIP8 function digging. By means of GO enrichment analysis, we filtered the top seven cellular components (CC), biological process (BP), and molecular function (MF) categories based on the following criteria: *p* value < 0.01 and FDR < 0.05. The data demonstrated that TNFAIP8 was linked to biological processes including death-inducing signaling complex, death receptor binding, and extrinsic apoptotic signaling pathway regulation (Figure 5A). This may explain the correlation between the TNFAIP8 higher expression level and better prognosis in SKCM. KEGG pathway analysis confirmed that apoptosis was the most significantly enriched pathway, and regulation of autophagy ranked second (Figure 5B). Autophagy is a key mechanism for inhibiting tumorigenesis (Qu et al., 2003). In macrophages, autophagy is essential for intratumoral invasion (Lin et al., 2013), while T-cell autophagy significantly enhanced functional CD8^+^ T-cell response and maintenance of tumor immune surveillance after tumor resection (Curry et al., 2017). The relevance between TNFAIP8 and autophagy made us interested in its role in immunity in SKCM.

### GSEA of TNFAIP8 in SKCM

**FIGURE 6:**
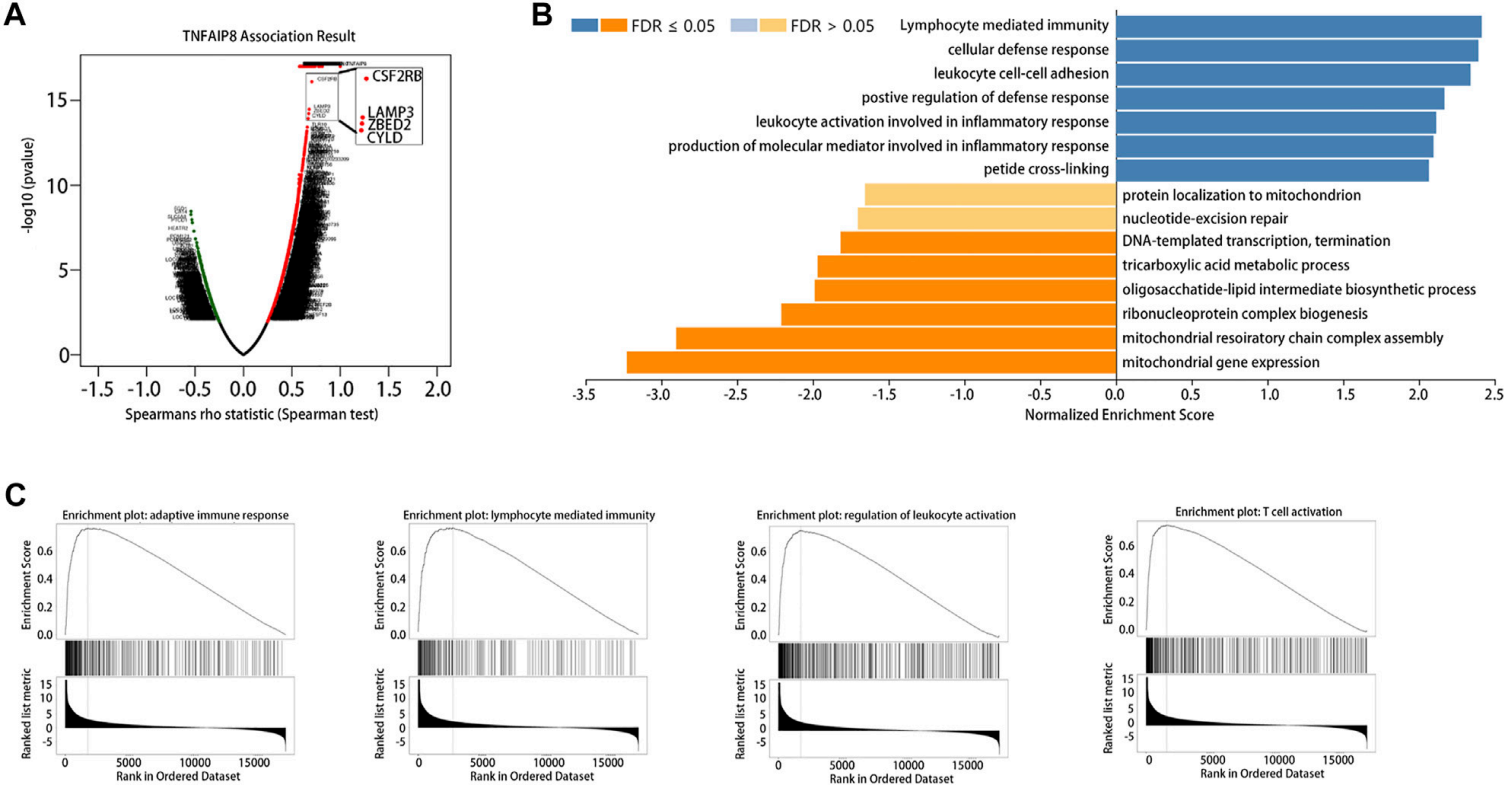
Gene set enrichment analysis (GSEA) of TNFAIP8 in SKCM. **(A)** Volcano plot of all differentially expressed genes. **(B)** Significantly enriched biological processes of GSEA annotations of TNFAIP8 in SKCM (LinkedOmics). **(C)** GSEA enrichment plots of specified biological processes.

The LinkedOmics database was used for gene set enrichment analysis (GSEA) of TNFAIP8 to investigate potential biological processes and pathways. We further examined TNFAIP8 co-expression genes in the SKCM cohort by LinkedOmics. The volcano plot indicated that 2089 genes (dark red dots) demonstrated mRNA expression levels that were positively correlated with the TNFAIP8 expression, whereas 389 genes (dark green dots) were negatively correlated with the TNFAIP8 expression (Figure 6A, FDR <0.01). Interestingly, GSEA of TNFAIP8 results showed TNFAIP8 relevant genes were enriched in immunoactivity including lymphocyte-mediated immunity, cellular defense response, positive regulation of defense response, and leukocyte activation involved in inflammatory response (Figures 6B,C). This is in line with the aforementioned result of PPI prediction that TNFAIP8 interacted with PTPRC and gave a better clue of TNFAIP8 in immune regulation. These pieces of evidence prompted us to conduct a subsequent study on the effect of TNFAIP8 on immune infiltration.

### Relationship Between TNFAIP8 and Immune Infiltration in the Tumor Microenvironment

**FIGURE 7:**
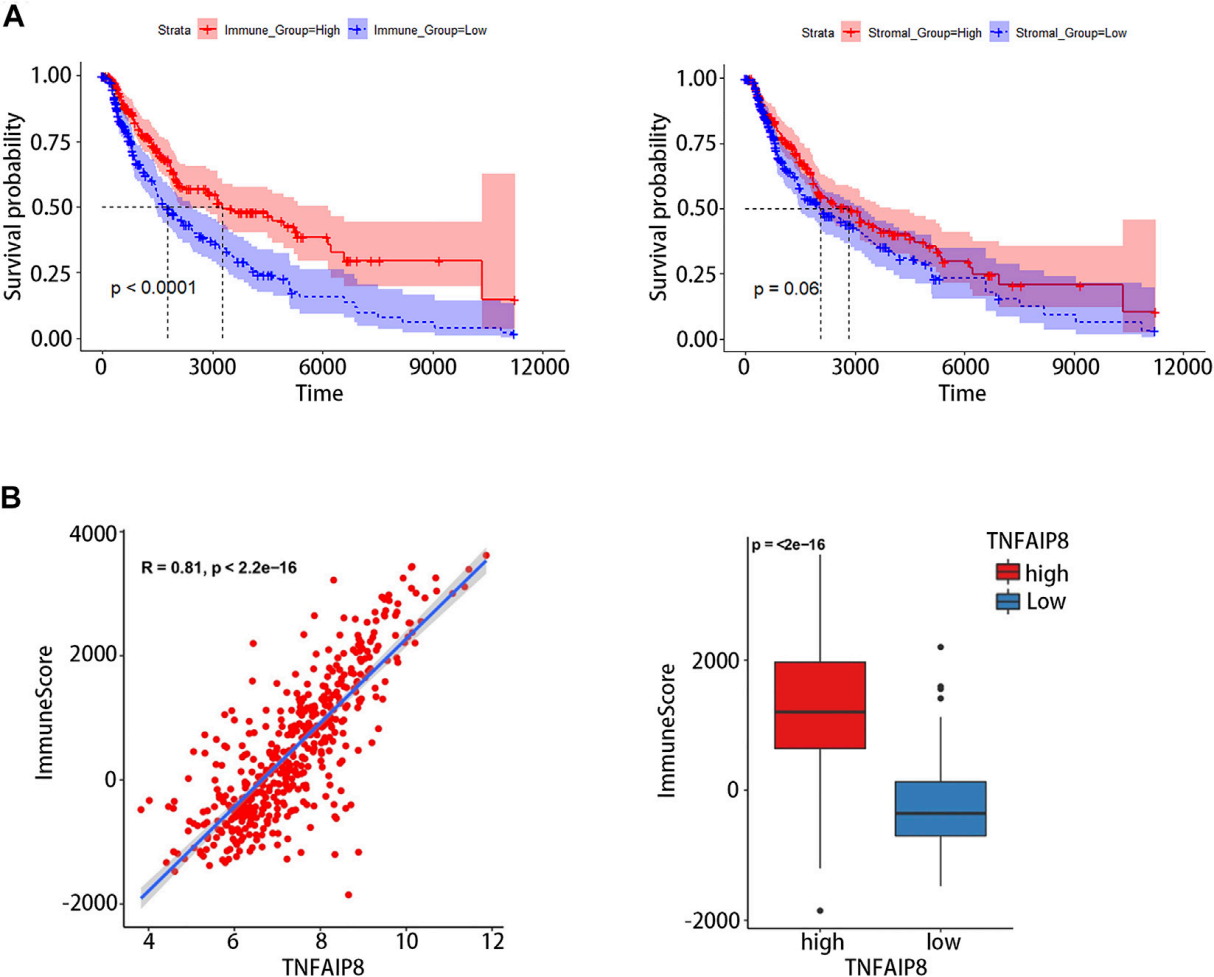
Correlation between the tumor immune environment (TME) and TNFAIP8 expression. **(A)** Correlation analysis between OS and the immune score **(left)** or stromal score **(right)**. **(B)** Correlation between TNFAIP8 and the immune score by a linear graph **(left)** or dichotomy graph **(right)**.

**FIGURE 8:**
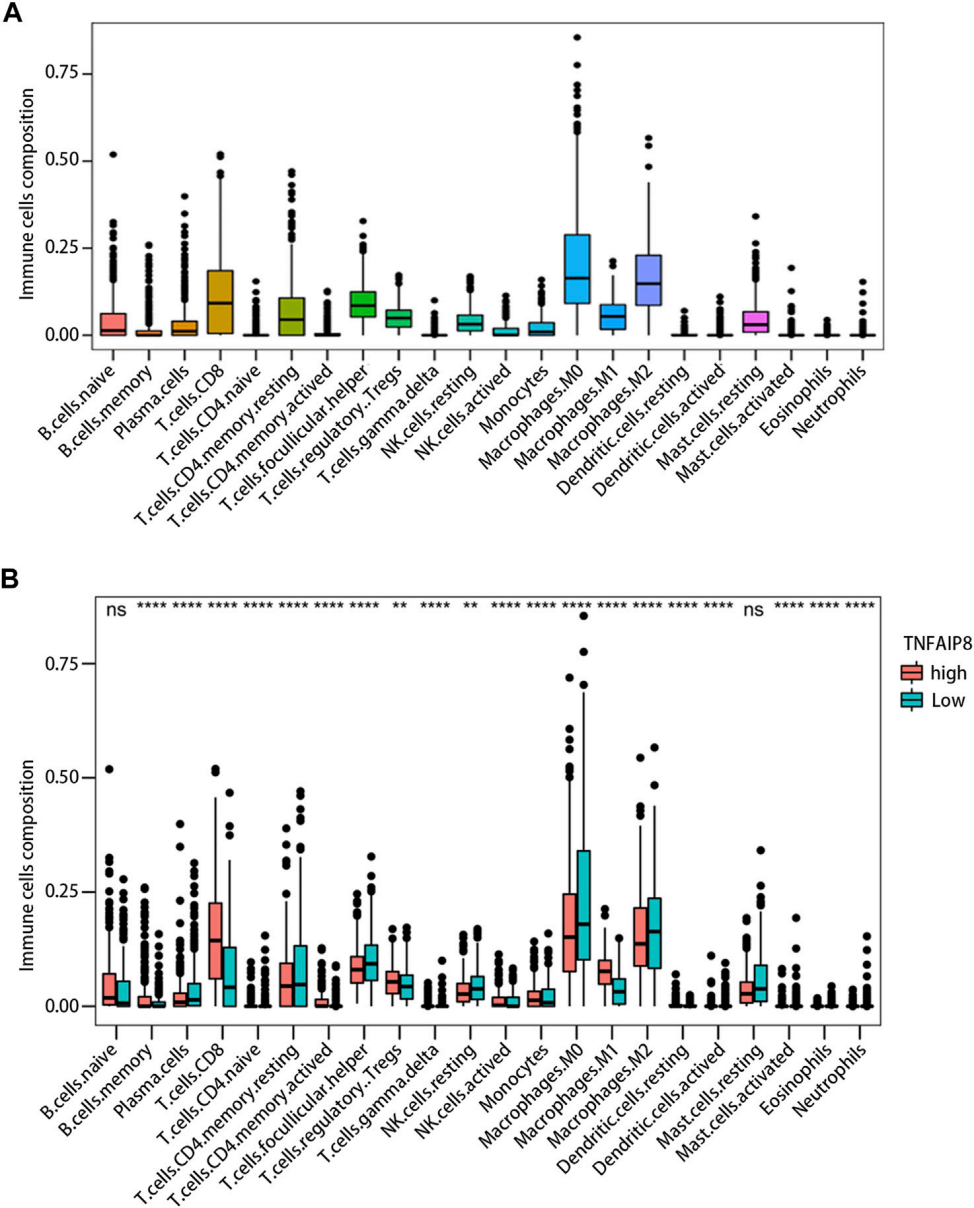
Impact of the TNFAIP8 expression on immune infiltration. **(A)** The proportion of 22 immune cell subtypes in SKCM. **(B)** Correlations between the TNFAIP8 expression and the infiltration abundances of selected immune cells in SKCM through the CIBERSORT algorithm. **p < 0.01; ****p < 0.0001; *ns*, no significant difference (*p* > 0.05).

Tumor-infiltrating lymphocyte grade has been shown to be an independent predictor of survival and sentinel lymph node status in patients with a variety of cancer types, including melanoma (Azimi et al., 2012; Oble et al., 2009). We calculated immune scores and stromal scores of SKCM and evaluated their impact on prognosis. The results showed high immune scores were significantly associated with an optimistic overall survival, while stromal scores were not related to prognosis (Figure 7A). The correlation between the immune score and TNFAIP8 mRNA expression was further analyzed. The expression of TNFAIP8 was positively correlated with the immune score, and a significant difference could be observed by dichotomization (Figure 7B). Association analysis between the TNFAIP8 expression and the level of immune-subgroups infiltration was further performed in the TCGA-SKCM cohort by R-package “*CIBRTSORT*.” The proportion of immune cell infiltration is shown in Figure 8A and divided into two groups according to the level of the TNFAIP8 expression (Figure 8B). In SKCM patients, high expression of TNFAIP8 significantly increased the proportion of CD8 T cells, CD4 memory T cells, M1 macrophages, and activated NK cells. By contrast, the high expression of TNFAIP8 significantly inhibited the proportion of immunosuppressive cells such as Tregs and M2 macrophages. The results show that the TNFAIP8 expression level has a significantly positive correlation with the immune infiltration level. These findings led a heavy hint that TNFAIP8 affects patient survival *via* interacting with immune infiltration in SKCM.

### Co-Expression Patterns Between TNFAIP8 Expression and Immune Checkpoints

**FIGURE 9:**
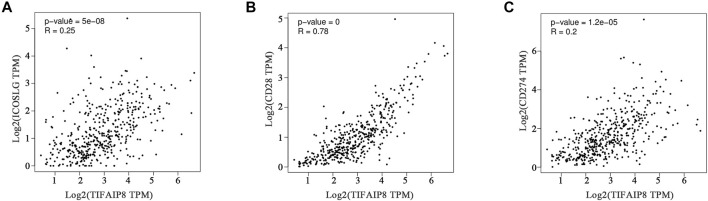
Correlation analysis between the TNFAIP8 expression and *ICOSLG* **(A)** or *CD28* **(B)** or *CD274* **(C)**.

Immune checkpoint therapy has become an important treatment for SKCM and has greatly improved the prognosis of patients (Curti and Faries, 2021). To further illuminate the correlation between the TNFAIP8 expression and immune checkpoint–targeted therapies, we explored the relation between the TNFAIP8 expression and immune checkpoint markers of SKCM. The gene mRNA expressions of *ICOSLG*, *CD28*, and *CD274* were selected. The results showed that all these six checkpoint markers were significantly co-related to the TNFAIP8 mRNA expression (Figure 9). This result suggests a possible mechanism that TNFAIP8 affects SKCM prognosis.

## Discussion

Previous studies have proved the expression pattern and importance of the TNFAIP8 family in biological systems. Most reports believe that TNFAIP8 and TIPE3 have antiapoptotic and tumorigenesis effects, while TIPE1 and TIPE2 have pro-apoptotic and antitumor effects in most cancers (Lou and Liu, 2011). The exogenous expression of TNFAIP8 promoted proliferation and migration in breast cancer. Higher levels of TNFAIP8 protein expression were found in human breast cancer and renal cell carcinoma tissues than in matched normal adjacent tissues (Kumar et al., 2004). The exogenous expression of TNFAIP8 promoted proliferation and migration in breast cancer both *in vitro* and *in vivo* (Zhang et al., 2006). The TNFAIP8 expression is elevated in a variety of cancer tumors, and a higher expression is associated with a lower survival rate of patients, suggesting that TNFAIP8 may play an important role in tumorigenesis as an oncogene. However, in TCGA data, different from most other tumor types, the low expression level of TNFAIP8 in SKCM relative to adjacent tissues attracted our attention. Compared with the TNFAIP8 high expression group, the low expression of TNFAIP8 was associated with poorer OS, suggesting that a high expression of TNFAIP8 could be regarded as a predictor of good prognosis of SKCM. This is contrary to the antiapoptotic and tumorigenic properties of TNFAIP8 that have been widely reported. Meanwhile, the TIPE2 expression was still associated with a better prognosis in SKCM, as reported in the literature. According to GO analysis, TNFAIP8 is enriched in a death-inducted signaling complex, death receptor binding, and other aspects related to cell death. KEGG pathway analysis also showed TNFAIP8 exhibits apoptosis promoting function. All of these results indicate the potential of TNFAIP8 as an antitumor marker in SKCM and can explain that the phenomenon of high expression of TNFAIP8 promises better prognosis.

Our study showed that the level of immune infiltration lymphocytes and various immune infiltration–related gene markers were positively correlated with the TNFAIP8 expression level. TNFAIP8 is highly expressed in the lymphoid tissue and placenta, suggesting that TNFAIP8 may play a role in the regulation of inflammation and immunity (Lou and Liu, 2011). Previous reports have shown that TNFAIP8 mRNA and TNFAIP8 protein levels are lower in lung tumor–infiltrating CD4^+^ and CD8^+^ T cells than in peripheral CD4^+^ and CD8^+^ T cells (Wang et al., 2014). In addition, the expression of TNFAIP8 in tumor-infiltrating CD8^+^ T cells in advanced lung cancer patients was lower than that in primary lung cancer patients, suggesting that the loss of TNFAIP8 may be involved in the progression of non–small-cell lung cancer (Wang et al., 2014).

Recently, we investigated the effect of TNFAIP8 on cell-mediated immunity of differentiated CD4^+^ T lymphocyte clusters in a mouse model (Yu et al., 2018). It was shown that the TNFAIP8 expression promoted proliferation of CD4^+^ T lymphocytes *in vitro*. The expression of TNFAIP8 also affects the polarization of splenic CD4^+^ T lymphocytes after sepsis, suggesting that TNFAIP8 regulates the pathogenesis of splenic T lymphocyte immune dysfunction in mice (Yu et al., 2018). Glucocorticoids are known to induce apoptosis and affect many human physiological systems, including the nervous, skeletal, muscular, endocrine, circulatory, and immune systems (Gruver-Yates and Cidlowski, 2013), and a recent study showed that TNFAIP8 promoted glucocorticoid-mediated apoptosis of mouse thymocytes (Woodward et al., 2010). It was found that the proliferation activity of CD4^+^ T lymphocytes was significantly downregulated 24 h after burn when the TNFAIP8 gene was silenced by siRNA in mice. These results suggest that TNFAIP8 appears to be involved in the immunomodulation of CD4^+^ T lymphocytes and that the decreased expression of TNFAIP8 may affect T lymphocyte function after heat injury. Therefore, the effect of TNFAIP8 on inflammation, immune function, and homeostasis on a variety of disease conditions has the potential to be further investigated.

The use of immunotherapy in SKCM has made a big success. It has been reported that the expression level of immune checkpoint proteins such as PD-L1 is related to the sensitivity of immune checkpoint blockade (Curti and Faries, 2021). Co-relation analysis showed that TNFAIP8 was highly correlated with the mRNA expression levels of immune checkpoint markers such as *CD274* (encoding PD-L1), indicating that TNFAIP8 may activate antitumor immunity and may serve as a prognostic indicator of SKCM treatment and PD-L1 therapy. Combined with the aforementioned positive correlation between TNFAIP8 and the level of immune infiltration, it is expected TNFAIP8 to become an indicator of PD-1-/PD-L1-related immune sensitivity and has certain clinical guiding significance.

There are some limitations of the analyses in this study. Associations between gene and clinicopathological polymorphism are lack of considerations. The analyses mentioned in this study are all association analyses, instead of causation analyses. It is expected to be further verified by experiments in the future. This study is a pioneer attempt, and further studies of these genes are required to provide a more comprehensive understanding of the potential relationship between the prognosis of TNFAIP8 and the tumor microenvironment. In conclusion, our study provides an opportunity to elucidate the underlying association between the TNFAIP8 expression and SKCM immune function, as well as its valuable potential as a prognostic biomarker for SKCM.

## Data Availability

Publicly available datasets were analyzed in this study. This data can be found here: https://portal.gdc.cancer.gov/ https://gtexportal.org/.

## Abbreviations

BP, biological processes; CC, cellular components; GEPIA, Gene Expression Profiling Interactive Analysis; GO, gene ontology; GSEA, gene set enrichment analysis; GTEx, Genotype-Tissue Expression Project; KEGG, Kyoto Encyclopedia of Genes and Genomes; MF, molecular functions; OS, overall survival; PAICS, phosphoribosylaminoimidazole carboxylase and phosphoribosylaminoimidazolesuccinocarboxamide synthase; PPI, protein–protein interaction; PTPRC, protein tyrosine phosphatase, receptor type C; SKCM, skin cutaneous melanoma; TNFAIP8, tumor necrosis factor-α–induced protein 8.
